# LOOPS for thought: identifying gastrointestinal pathology on transthoracic echocardiography

**DOI:** 10.1093/ehjcr/ytag339

**Published:** 2026-05-08

**Authors:** Ahmed Magdy, Reza Arsanjani, Tasneem Naqvi

**Affiliations:** Cardiovascular Disease Department, Mayo Clinic, 5881 E. Mayo Blvd., Building 3, Phoenix, AZ 85054, USA; Cardiovascular Disease Department, Mayo Clinic, 5881 E. Mayo Blvd., Building 3, Phoenix, AZ 85054, USA; Cardiovascular Disease Department, Mayo Clinic, 5881 E. Mayo Blvd., Building 3, Phoenix, AZ 85054, USA

**Keywords:** Transthoracic echocardiography, Hiatal hernia, Oesophageal varices, Extracardiac structures, Gastrointestinal pathology, Case report

## Abstract

**Background:**

Transthoracic echocardiography (TTE) is the most commonly used modality for the diagnosis and follow-up of cardiac diseases. During routine TTE, surrounding extracardiac structures are often visualized, including the lungs, pleural spaces, oesophagus, diaphragm, and sub-diaphragmatic structures such the liver, stomach, bowel, and abdominal aorta. Pathologic conditions affecting these organs may exert extrinsic compression on the cardiac chambers, leading to diagnostic uncertainty or mimicking cardiac disease by producing symptoms, such as dyspnoea, fatigue, chest pain, or palpitations.

**Case summary:**

We describe five patients who underwent routine TTE and were incidentally found to have unsuspected gastrointestinal abnormalities. The findings subsequently confirmed by other imaging modalities included hiatal hernia, distended bowel loops, oesophageal carcinoma, and oesophageal varices. Reporting of these findings impacted the diagnosis and management of the cases.

**Conclusion:**

Awareness of the sonographic appearance of gastrointestinal during TTE is essential, as these findings may represent clinically significant pathology that mimics or contributes to cardiac symptoms. Recognition of such findings may be enhanced by modified echocardiographic views, with confirmatory evaluation using chest radiography, computed tomography, or gastrointestinal endoscopy as appropriate

Learning points

- Awareness of the sonographic appearance of gastrointestinal and other extracardiac abnormalities during transthoracic echocardiography (TTE) is essential
- The best TTE views to detect are usually parasternal long-axis, apical four-chamber, and subcostal views. Modified views might be needed.
- Reporting and confirmatory evaluation with chest X-ray, computed tomography, or gastrointestinal endoscopy is recommended.

## Introduction

The heart lies in close proximity to several gastrointestinal (GI) structures: the oesophagus is positioned posterior to the left atrium (LA), while the liver and stomach are separated from the inferior wall of the heart by the diaphragm. The GI tract may exert external compression on one or more cardiac chambers. These can result in non-specific symptoms, such as dyspnoea, fatigue, palpitations, and chest pain and may even mimic acute coronary syndrome from electrocardiogram (ECG) changes related to electrolyte imbalance or from changes in cardiac axis.^1^

These extracardiac structures can often be visualized during routine transthoracic echocardiography (TTE) from various imaging windows. Careful recognition and systematic assessment of these findings may provide critical diagnostic clues for significant as well serious underlying GI pathologies.^2^ Optimized echogenic windows, as well as the use of standardized and modified TTE views, may be required to adequately evaluate extracardiac, particularly GI structures.

We present a case series of five patients who underwent TTE, either for evaluation of cardiac-related symptoms, such as dyspnoea and fatigue, or as a part of routine assessment for non-cardiac diseases and had incidentally detected extracardiac oesophageal, gastric, and intestinal GI pathology. Our cases highlight characteristic echocardiographic features, diagnostic pitfalls, downstream testing, and clinical implications.

## Summary figure

**Figure:**
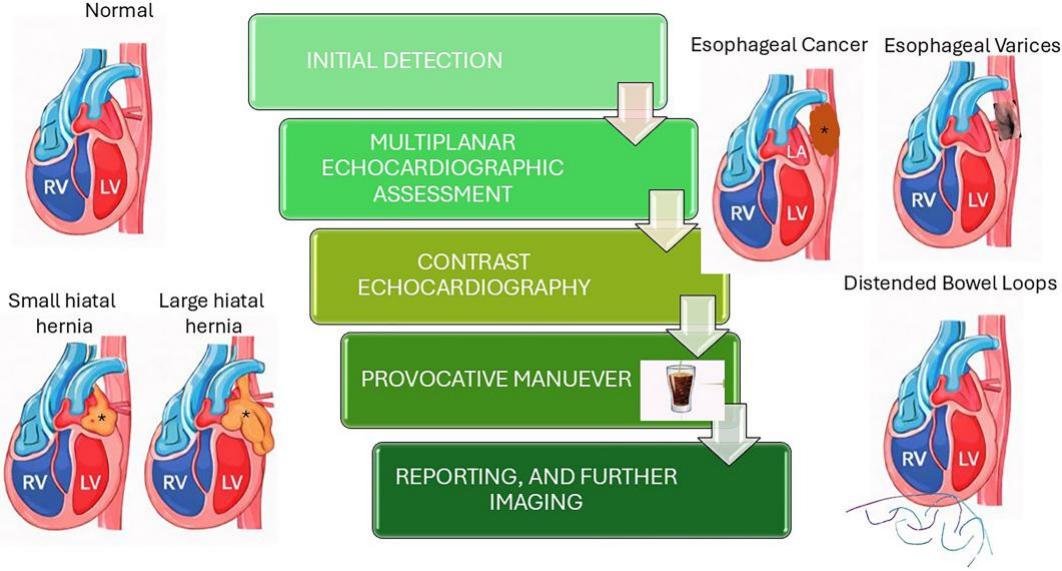
The diagram shows the relationship of the left heart with the oesophagus, stomach, liver, and intestines. Normal structures from the lateral view are shown on the top left. Oesophageal cancer posterior to the LA and left ventricle and oesophageal varices posterior to the LA and left ventricle are shown in the top R panel. Progressively larger hiatal hernias pushing on the basal LV posterolateral wall and posterior wall of the LA are shown in the bottom left panel. Distended intestinal loops are shown in the bottom right panel. Extrinsic LA or posterior LV wall compression in the PLAX view, including pseusodyskinesis of the LV posterior wall, should alert for the presence of extracardiac or subdiaphragmatic pathology. The structure should be pursued in short-axis, apical, and subcostal views, including off-axis views if needed. The use of microbubble enhancement may help exclude intracardiac mass. Carbonated drink may allow visualization of bubbles in a hiatal hernia. Comment should be made regarding ECF in the echo report with recommendation for further imaging including abdominal ultrasound, CXR, chest or abdominal CT. LV, left ventricle; RV, right ventricle; LA, left atrium.

## Case 1

**Figure 1:**
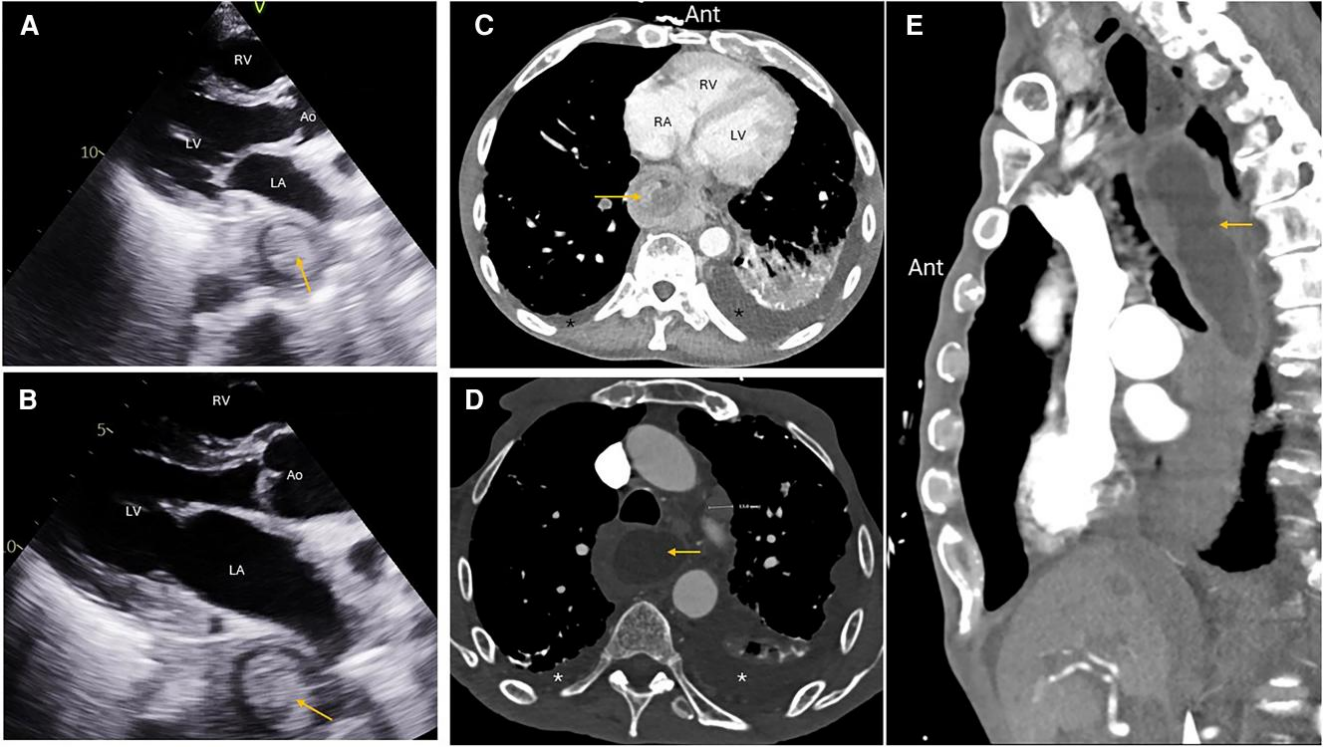
Parasternal long-axis views in systole (*A*) and diastole (*B*) showing a well-circumscribed spherical echo dense mass (arrow) posterior to the left atrium causing extrinsic left atrium compression in systole (*A*). Transverse computed tomography chest views at the cardiac (*C*) and upper thoracic (*D*) levels showing the same mass in the oesophagus (arrows) posterior to the left atrium (*C*) and the trachea (*D*). Left pulmonary fibrosis and pleural effusions (black asterisks *C*, white asterisks *D*). (*E*) A coronal computed tomography chest view showing the mass in a dilated oesophagus. The mass was an oesophageal adenocarcinoma on biopsy. LV, left ventricle; LA, left atrium; RV, right ventricle; Ao, aorta; RA, right atrium; Ant, anterior.

A 71-year-old male with a history of metastatic oesophageal adenocarcinoma complicated by GI bleeding and pulmonary embolism following deep venous thrombosis, status post-inferior vena cava filter placement, was evaluated. His medical history was further notable for respiratory failure and chronic obstructive pulmonary disease with associated pulmonary hypertension. A TTE was performed to assess his cardiac status. On the PLAX view, a solid, rounded heterogeneous mass measuring 3.5 × 3.1 cm was identified posteriorly, exerting mild mass effect on the LA. A differential diagnosis of benign vs. malignant oesophageal mass or a metastatic tumour was made. Subsequent abdominal CT confirmed the finding, demonstrating a large enhancing soft tissue mass involving the lower oesophagus and extending into the oesophagogastric junction and proximal stomach (*Figure 1*).

## Case 2

**Figure 2:**
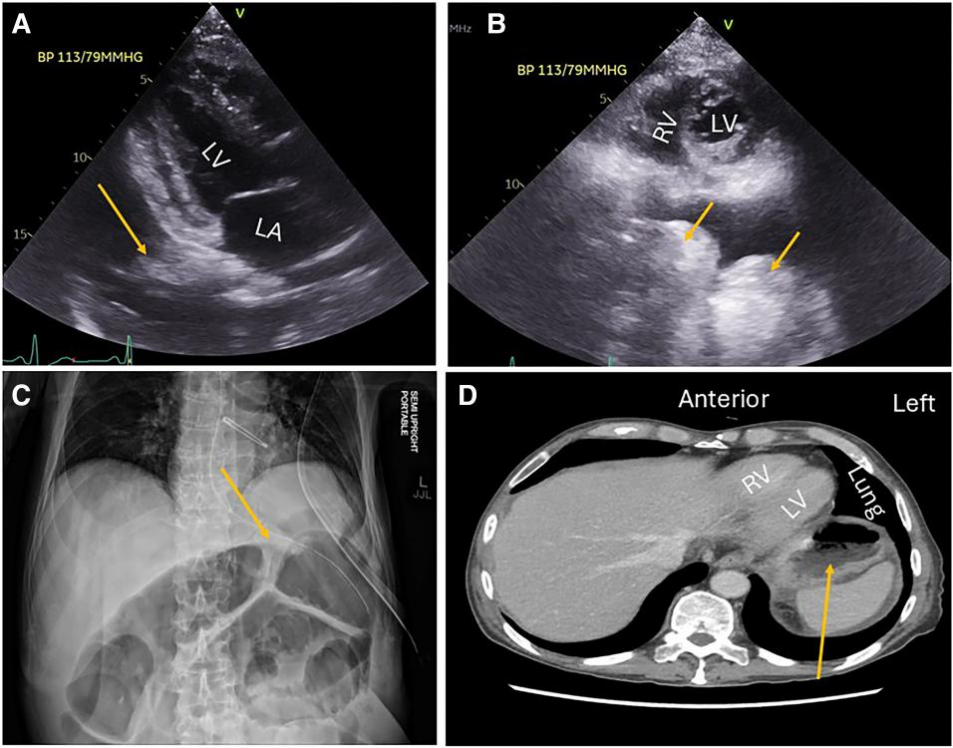
Apical three-chamber view (*A*) showing a bright structure (arrow) related to the posterior wall of the left atrium. Parasternal short-axis view (*B*) showing two large bright white space occupying structures (arrows) related to the posterior wall of the left ventricle at the mid-cavitary level. X-ray (*C*) showing the lower chest and upper abdomen with severely distended bowel. Transverse computed tomography scan (*D*) of the upper abdomen showing distended bowel loops (arrow). LV, left ventricle; LA, left atrium; RV, right ventricle.

A 71-year-old male with a history of inflammatory bowel disease (IBD), complicated by enteroenteric fistulas and multiple prior GI surgeries and interventions, was admitted with acute exacerbation of chronic IBD. During his hospitalization, TTE demonstrated an echolucent space and distended bowel loops abutting the posterior wall of the heart suggestive of possible free air or fluid with bowel perforation. Subsequent chest and abdominal CT imaging showed distended and oedematous loops of the small bowel in the upper abdominal cavity (*Figure 2*).

## Case 3

**Figure 3:**
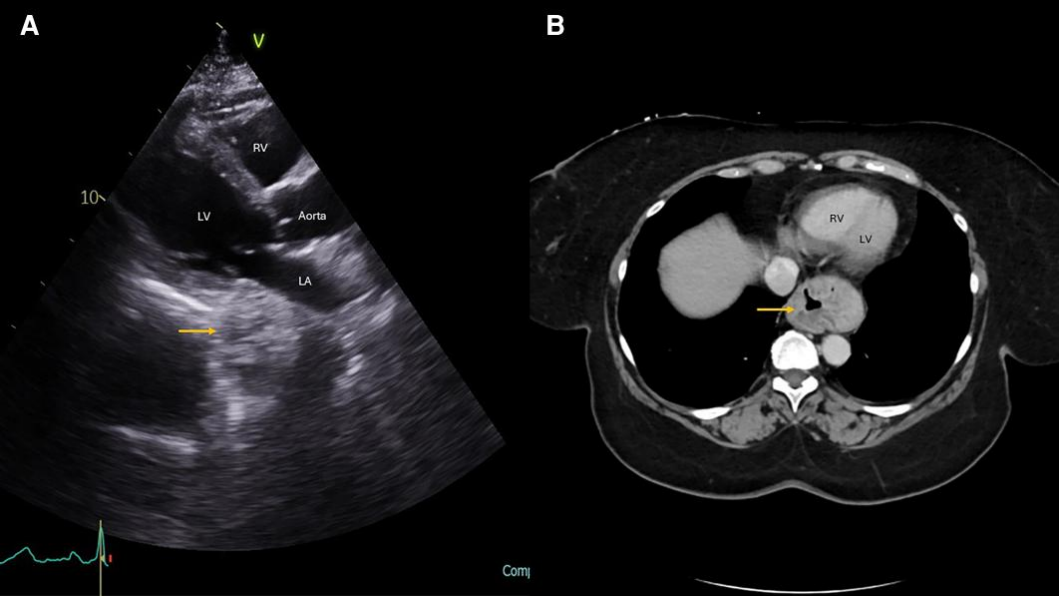
Parasternal long-axis view (*A*) showing a rounded heterogeneous echo-dense mass (arrow) flattening the posterior wall of the left atrium and appearing to be within the left atrium in the apical views (not shown). Computed tomography chest transverse view (*B*) showing the mass (arrow) posterior to the left atrium with black space indicating air. The diagnosis revealed a medium-sized hiatal hernia. LV, left ventricle; LA, left atrium; RV, right ventricle.

A 67-year-old female presented with complaints of dysphagia. A TTE was performed as part of routine evaluation. On the PLAX view, a rounded somewhat ill-defined echogenic mass was seen located posterior to the LA, causing mild extrinsic compression. Subsequent abdominal CT confirmed the presence of a moderate hiatal hernia (*Figure 3*). The echocardiography here helped identify the cause of dysphagia due to GI reflux and esophagitis.

## Case 4

**Figure 4:**
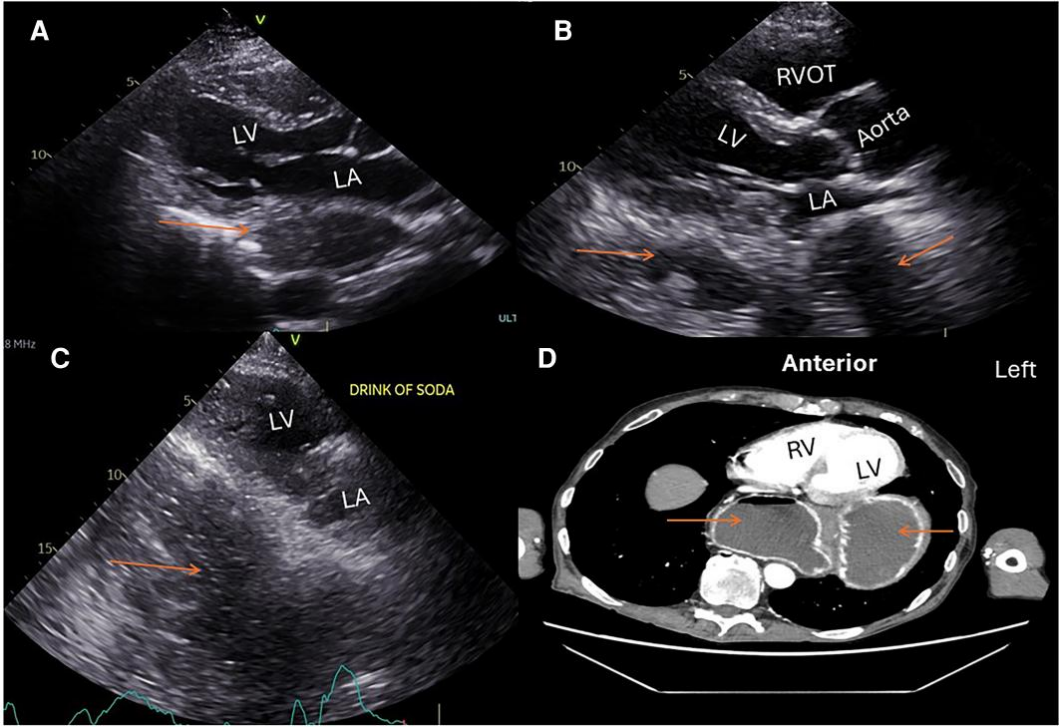
Parasternal long-axis view (*A* and *B*) showing a large structure (arrow) posterior to the left atrium causing extrinsic left atrium compression (*A*). Parasternal long-axis view (*C*) showing the structure posterior to the left atrium filled with soda bubbles (arrow) immediately after soda drink. Transverse computed tomography chest view at the cardiac level (*D*) showing the same structure with air level (arrows) posterior to the left atrium. The structure was part of the stomach in a patient with hiatal hernia. LV, left ventricle; LA, left atrium; RVOT, right ventricle outflow tract.

A 90-year-old male with a history of hypertension, dyslipidaemia, non-obstructive coronary artery disease, advanced stable ulcerative colitis, and laparoscopic cholecystectomy presented for a routine TTE. He had a recent diagnosis of gastroparesis after a Whipple procedure and Roux-en-Y gastrojejunostomy. On the parasternal long-axis (PLAX) view, there was extrinsic invagination of the left atrial and adjacent left ventricular (LV) inferolateral wall from an echogenic ill-defined circular mass with ‘sparkling’ bubbles (*Figure 4A*). Administration of a carbonated beverage during the study enhanced microbubbles suggesting the presence of a hiatal hernia, as the passage of air bubbles into the herniated stomach was readily detected on echocardiography (*Figure 1C*). The diagnosis was subsequently confirmed by chest and abdominal computed tomography (CT) imaging (*Figure 1D*) as well as upper GI endoscopy, both of which documented the presence of a hiatal hernia.

## Case 5

**Figure 5:**
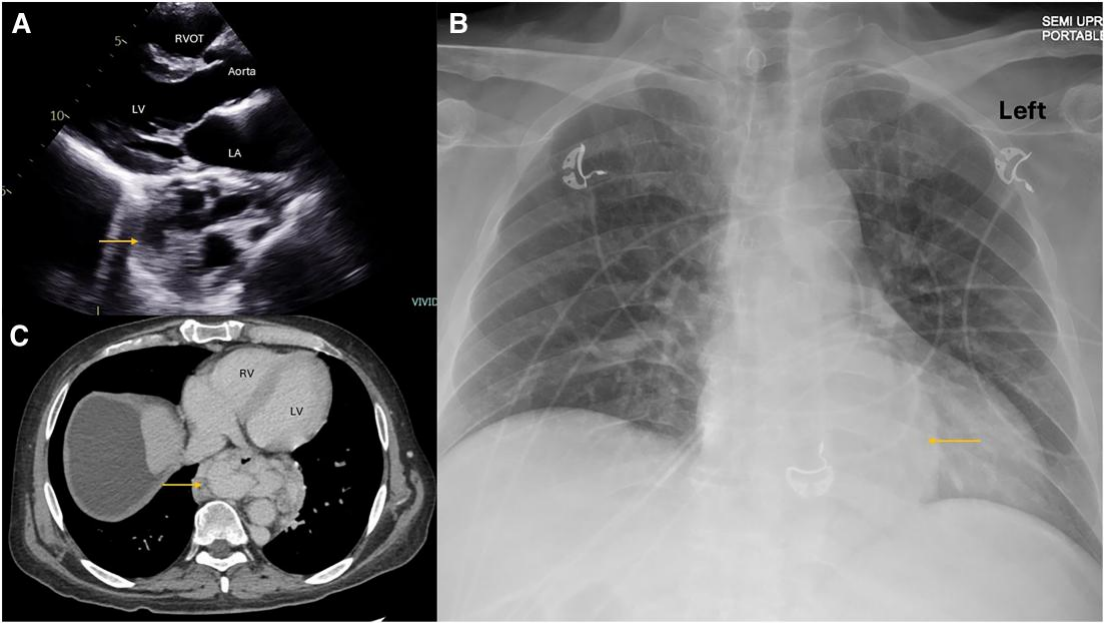
Parasternal long-axis view (*A*) showing a large well-circumscribed mass with cavitations posterior to the left atrium and basal left ventricle. Antero-posterior chest X-ray (*B*) revealed a large, rounded mass (arrow) located behind the cardiac silhouette. Computed tomography chest transverse view (*C*) showing a large multinodular-appearing mass (arrow) behind the left atrium and basal left ventricle. These are large oesophageal varices in a patient with hepatic cirrhosis and porto-pulmonary hypertension. LV, left ventricle; LA, left atrium; RVOT, right ventricle outflow tract.

A 53-year-old male with a history of decompensated cirrhosis presented 6 months post liver transplantation for routine follow-up. Transthoracic echocardiography demonstrated a large, circumscribed mass posterior LA with multiple rounded echolucencies in the PLAX view (*Figure 5A*). Colour Doppler imaging was not performed. Differential diagnoses for small cavitary lesions within a contact sac included a large necrotic tumour, a fungal mass, hydatid cyst, and a haemangioma. Chest CT revealed ill-defined soft tissue mass in the lower paraoesophageal mediastinum with large oesophageal varices (*Figure 5*).

## Discussion

This case series illustrates that routine TTE can incidentally identify clinically relevant extracardiac GI pathology involving the upper, mid-, and lower GI tract that were unexpected, helped explain patients’ symptoms, and led to further diagnostic workup. These findings were primarily visualized from standard parasternal, subcostal, and apical views. A large observational study of 41 067 echocardiographic studies in a large health network demonstrated that extracardiac findings are reported in ∼4.4% of echocardiograms: pleural effusions being the most common finding on TTE and aortic atheroma on TEE. Notably, abdominal and GI-related findings are more likely than pleural abnormalities to prompt additional imaging and new diagnoses, underscoring their relevance.^3^ Certain GI pathologies may manifest as cardiac symptoms, either through direct mechanical compression of cardiac chambers or by mimicking cardiopulmonary pathology. In such cases, TTE, particularly when performed in patients with favourable echogenic windows, can provide the first diagnostic clue to an underlying serious GI condition. Careful attention to extracardiac structures during routine echocardiographic evaluation is therefore essential.

Parasternal long-axis image oftentimes provides the first clue as the extrinsic compression is first identified posterior to LA and LV. It may at times be confused with an intracardiac mass within the LA. Changes and motion of the mass with respiration may be an important clue to its extracardiac location.^4^ Pseudo-dyskinesis of the LV inferolateral wall from elevated left hemidiaphragm may offer clues to the presence of diaphragmatic or subdiaphragmatic pathology. Another way to differentiate extracardiac from LA masses in cases with LA compression is contrast echocardiography. That allows better demarcation of the cardiac borders with the extracardiac structures not filling with contrast.

Hiatal hernia represents the most frequently encountered and well-described extracardiac entity that may appear as a retrocardiac echolucent or mixed-density structure adjacent to the LA, occasionally mimicking an intracardiac mass.^3^ Recognition of this appearance is essential to avoid misdiagnosis and unnecessary invasive investigations. Administration of a carbonated beverage can help enhance visualization of GI contents, facilitating recognition of a hiatal hernia.^5^ Its size can vary considerably, as illustrated in Patients 3 and 4. In rare cases, it can exert mass effect on cardiac chambers. A rare case with huge hiatal hernia causing left atrial compression with significant haemodynamic instability with emergent aspiration of the gastric content and surgical repair afterwards was reported in 2021.^6^ Evaluation from multiple echocardiographic windows, including off-axis views, is often required to confirm the extracardiac origin of a mass-like structure and to exclude an intra-atrial lesion. In many patients, subcostal windows provide best windows for distended bowel with fluid contents with or without ascites.

Correlation with other imaging modalities—such as chest radiography, CT of the chest or abdomen, or GI endoscopy if available at the time of TTE interpretation—can further aid in identifying confusing extracardiac masses and prevent misdiagnosis.^3^ Reporting of these findings in Cases 4 and 5 was valuable in rearranging the order of other investigations as the treating physicians started with CT before GI endoscopy to get better understanding of the mediastinal anatomy before the endoscopy. Diagnosis of hiatal hernia is especially useful since GI reflux often manifests as chest pain and early diagnosis may reduce diagnostic workup.

Identification and appropriate reporting of suspicious extracardiac findings may expedite targeted abdominal imaging and specialist referral. However, detection of extracardiac findings is inherently operator and interpreter dependent and may be underestimated. Our cases highlight the importance of systematic inspection of extracardiac structures during comprehensive TTE and support inclusion of a dedicated ‘extracardiac findings’ section in echocardiography reports.^3^ Future studies should focus on developing consensus reporting standards and evaluating the cost-effectiveness of structured extracardiac assessment during echocardiography.

## Data Availability

All patient data have been presented in this report.

## Lead author biography

Dr Ahmed Magyd is an advanced echocardiography fellow in Mayo Clinic Arizona. His main interest is cardiovascular imaging with specific interest in echocardiography modalities, including TTE and TEEs. He has a dual postgraduate degree with MSc in cardiology and MPH.

## Notes

**Consent:** Written informed consent was obtained from the patients for publication of this case series and any accompanying images.
